# Turkish Validity and Reliability Study of the Short Version of the Questionnaire for Assessing the Childbirth Experience (QACE)

**DOI:** 10.3390/healthcare13141743

**Published:** 2025-07-18

**Authors:** Cevriye Emir, Candan Ozturk

**Affiliations:** 1 Institute of Graduate Studies, Near East University, Lefkosa KKTC, Mersin 10, 99138 Nicosia, Turkiye; 2 Faculty of Nursing, Near East University, Lefkosa KKTC, Mersin 10, 99138 Nicosia, Turkiye; candan.ozturk@neu.edu.tr

**Keywords:** childbirth experience, measures, reliability, validity, QACE

## Abstract

**Objective:** The mother’s birth experience is crucial in identifying her care needs. The “Questionnaire for Assessing the Childbirth Experience” (QACE) consists of four subscales designed to assess a mother’s childbirth experience. However, psychometric evaluations of the QACE have not been conducted in Turkey. This study aims to adapt and assess the validity and reliability of the “Questionnaire for Assessing the Childbirth Experience” short version for the Turkish maternal population. **Methods:** This methodological study included 205 mothers who gave birth in a public hospital. Data were collected using an Introductory Information Form and the Questionnaire for Assessing the Childbirth Experience (QACE). **Results:** The content validity index (CVI) for Item 9 was calculated as 0.875, while the remaining 12 items obtained a CVI of 1.00. The internal consistency of the scale, as measured by Cronbach’s alpha coefficient (α = 0.758), indicated an acceptable level of reliability across all items. During the Exploratory Factor Analysis (EFA), one item (Item 13) was excluded due to cross-loading. The remaining 12 items were grouped into four distinct subscales. **Conclusions:** The short version of the QACE demonstrates acceptable psychometric properties and serves as a valid and reliable instrument for assessing childbirth experiences among Turkish women.

## 1. Introduction

Childbirth is a profoundly personal experience involving subjective psychological and physiological processes influenced by environmental, organizational, and political contexts [1]. During childbirth, women require care that addresses their specific needs, ensures continuity, and is based on their preferences and values [2]. Each mother’s birth process brings unique emotions, reactions, and challenges. Difficulties such as inability to breastfeed, reduced affection toward the baby, emotional distress, post-traumatic stress disorder, and depression reflect the complex dynamics of childbirth [3,4]. Mothers should be closely monitored for mental health during and after childbirth. Their emotional fluctuations should be addressed according to their needs [5]. The World Health Organization (WHO) emphasizes the prevalence of mistreatment during childbirth and its negative impact on mothers’ birth experiences, highlighting the importance of respectful and woman-centered care strategies [6].

A history of negative birth experiences, along with factors such as misinformation and fear of childbirth, may influence women to prefer elective cesarean sections without medical indications, thereby potentially undermining the natural course of childbirth [7,8]. Implementing childbirth education programs during pregnancy and providing spousal support during labor may increase maternal self-efficacy and enable women to manage labor pain more effectively [9,10].

In Turkish culture, childbirth is considered a sacred duty that every woman should experience. It is closely linked to religious practices and is believed to contribute to a woman’s maturation while shaping her perceptions of pain [11,12]. Recent reviews of clinical protocols implemented in Türkiye reveal an increase in caesarean rates and the frequent use of medical interventions during labor.

These developments have led to negative birth experiences and have triggered both short- and long-term complications affecting the health of mothers and babies [13,14,15,16]. All these factors emerge as multidimensional elements influencing the childbirth experience and must be understood within their cultural context. Therefore, it is crucial to develop or adapt tools that measure Turkish women’s childbirth experiences to reflect both cultural and systemic specificities, and to enhance healthcare professionals’ knowledge and skills regarding cultural sensitivity, woman-centered care strategies, pain perception, and the implementation of informed interventions, as these are essential for protecting maternal and baby health.

Identifying the multidimensional factors influencing childbirth is critical for concept analysis and developing effective care strategies [17,18,19,20]. Establishing these strategies on a solid foundation requires valid and reliable measurement of these factors. Modern assessment tools with strong psychometric properties support evidence-based, woman-centered care and improve quality and patient satisfaction [21,22,23]. The Questionnaire for Assessing the Childbirth Experience (QACE) was developed by Carquillat and colleagues (2017) based on a qualitative study conducted by Guittier et al. (2014). The researchers identified several key themes through their qualitative analysis, including “maternal emotions”, “first moments with the newborn”, and “staff attitudes and behaviors”, which were considered potentially influential in the development of the QACE [20,23]. QACE has been adapted to various cultural contexts, and its validity and reliability have been tested accordingly. The questionnaire has been successfully adapted in Iran [24], France [25], Spain [26], and the Chinese cultural context [27].

The main feature distinguishing this questionnaire from other measurement tools is that it includes four subscales, has a limited number of items, and focuses exclusively on the intrapartum period. For example, the Wijma Delivery Expectancy/Experience Questionnaire covers prenatal and postnatal periods [28]. In contrast, the Childbirth Experience Questionnaire (CEQ) contains more items, possibly requiring more time to administer [29]. In this context, the brief nature of the questionnaire and its focus on a specific period suggest that adapting it to Turkish culture may be beneficial. When evaluating instruments developed or adapted into Turkish to assess childbirth experiences, it has been observed that the dimensions are limited, and important aspects such as “relationship with healthcare staff”, “fırst moments with the newborn”, and “feelings at one month postpartum” are not addressed. Furthermore, there appears to be no instrument that encompasses all the factors related to the childbirth experience [30,31,32].

In the systematic review conducted by Cheng et al. (2024), eleven instruments measuring childbirth experience were evaluated using the COSMIN (Consensus-based Standards for the Selection of Health Measurement Instruments Risk of Bias Checklist) guidelines. The review concluded that the QACE’s methodological and psychometric properties were of a higher standard than those of the other instruments [33].

According to the results of the literature review, a valid and reliable measurement that comprehensively assesses the childbirth experiences of Turkish mothers to support the development of responsive care strategies, a method that incorporates all dimensions, is essential when evaluating the experiences of mothers who give birth.

## 2. Methods

### 2.1. Study Aim and Design

This methodological study aims to evaluate the psychometric properties of the “Questionnaire for Assessing the Childbirth Experience”, adapt it to Turkish culture by examining its validity and reliability, and introduce it into the Turkish literature.

### 2.2. Evaluating the Recommendations of the Expert Team to Determine the Language Expression Suitability and Content Validity of QACE

Before starting the adaptation study, the QACE’s development stages, structure, and psychometric properties were examined, and permission via email from the researchers who developed the questionnaire was obtained.

Before the adaptation study, the QACE’s development, structure, and psychometric features were reviewed, and official permission was obtained via e-mail from the questionnaire’s original developers.

Two experts proficient in both languages initially translated the questionnaire from English to Turkish. Subsequently, two bilingual experts who had not seen the original version back-translated it into English.

Eight nursing faculty members reviewed the Turkish version of the scale to assess the linguistic clarity and content validity. The experts were asked to evaluate each item in terms of language and clarity using the following rating scale:

1: Not appropriate
2: Requires major revision
3: Requires minor revision
4: Appropriate

Following the evaluations, the Content Validity Index (CVI) was calculated using the Davis method. The CVI for each item was determined by dividing the number of experts who rated it as 3 or 4 by the total number of experts. A CVI of 0.80 or above for each item was considered acceptable [34]. In this study, the CVI for item 9 was 0.875, while the remaining twelve items received a CVI of 1.00.

Additionally, feedback was obtained from Dr. Guittier Marie-Julia, one of the original developers of the QACE scale, and her recommendations were incorporated. As a result, the Turkish version of the questionnaire was confirmed to be linguistically and conceptually appropriate and culturally relevant.

After establishing content validity, a pilot study was conducted with 10 women who had given birth within one to one and a half months in the obstetrics and gynecology service of a public hospital in Northern Cyprus and shared similar characteristics with the target sample. The pilot results indicated no issues with the questionnaire items, and no revisions were needed before data collection.

### 2.3. Ethical Considerations

Before starting the research, written permission to adapt and use QACE in Turkish was obtained via email from Marie-Julia Guittier, and the study was conducted following the Declaration of Helsinki.

This study was approved by the Ethics Committee of the the State Hospital (Approval no YTX.1.01 04/23 dated 1 March 2023).

During the data collection, women were given brief information appropriate to the research purpose, the confidentiality principle was explained, and they were asked to sign a “Consent Form”, stating that their participation in the study was voluntary and that they could refuse to participate if they did not want to.

### 2.4. Measurements/Instruments

The data were collected using the “Introductory Information Form” and the “Questionnaire for Assessing the Childbirth Experience”.

#### 2.4.1. Introductory Information Form

İncludes socio-demographic and obstetric questions such as mothers’ age, education level, marital status, occupation, number of births, history of complicated childbirth, and mode of delivery. It was developed based on a literature review [23,24,25,26,27].

#### 2.4.2. Questionnaire for Assessing the Childbirth Experience

**Figure 1:**
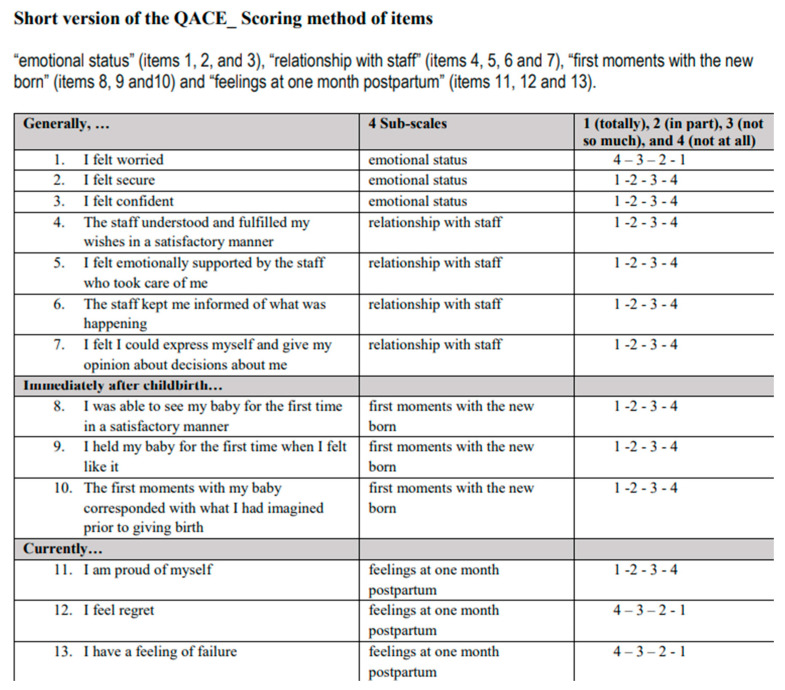
Short Version of QACE.

The Questionnaire for Assessing the Childbirth Experience (QACE) was developed by Carquillat, Vendittelli, Perneger, and Guittier (2017) to assess mothers’ birth experiences. The original language is French, and the original article presents the English form. The scale has a 25-item general form and a 13-item short form. In order to measure the construct validity of the general form of QACE, the responses to the items evaluating the birth experience were compared by rating them on a numerical scale of 0–10, and the descriptive analysis of the distribution of responses for the items showed a breakpoint at 8. The Numerical Assessment Scale scores were converted to categorical values to compare the two groups (“NRS scores of 0 to 7” and “NRS scores of 8 to 10”). The short version of QACE includes four subscales: “emotional status” (items 1, 2, and 3), “relationship with staff” (items 4, 5, 6 and 7), “first moments with the newborn” (items 8, 9 and 10) and “feelings at one month postpartum” (items 11, 12 and 13). The score range for each dimension is 1–4, with higher scores indicating negative experiences. To analyze the score data, the response format of the 4-point Likert scale was coded as 1 (totally), 2 (in part), 3 (not so much), and 4 (not at all) for items 2, 3, 4, 5, 6, 7, 8, 9, 10, and 11, with higher scores reflecting a more negative birth experience. The ratings of negatively worded statements were reverse-scored (Items 1, 12, and 13). Scores for each of the four dimensions are calculated as the average of the scores of the included items (Figure 1). A total score may not be calculated. Cronbach’s alpha coefficients for the sub-dimensions were given as 0.85 for “Relationship with staff”, 0.70 for “Emotional state”, 0.84 for “First moments with the newborn”, and 0.73 for “Emotions in the first month after birth”, respectively. QACE can be used as a research tool, and its short version (scoring according to subscales) and general version (per-item analysis) can be included in clinical practice. This study examined the validity and reliability of the short version of the scale in Turkish [23].

### 2.5. Data Collection

The study sample consisted of primiparous and multiparous mothers over 18 years who understood and spoke Turkish and gave birth in the obstetrical and gynecology service of a public hospital in Northern Cyprus. The mothers included in the study were selected by a simple random sampling method. The data was collected in this hospital between March and December 2023. “G. Using the “Power-3.1.9.2” program, the sample size was calculated at a 95.0% confidence.

The analysis revealed a standardized effect size of 0.27 at the α = 0.05 level, consistent with previous studies. The minimum sample size was calculated as 172 with a theoretical power of 0.95, and 205 mothers voluntarily participated.

### 2.6. Research Inclusion Criteria

(a) Women who consented to participate in the study;
(b) Having given birth within one to three months;
(c) Reading, speaking, and understanding Turkish;
(d) The mother must be 18 years or older;
(e) Gave birth at 37 weeks or more of pregnancy;
(f) Single and uncomplicated pregnancy.

### 2.7. Research Exclusion Criteria

(a) Presence of mental illness in the mother;
(b) Birth under 18 years of age; (c) Mother and baby are separated after birth for medical reasons; (d) Completing the pilot application process.

Data were collected using face-to-face and telephone interview techniques from 205 mothers who gave birth within one to one and a half months in the obstetrics and gynecology service of a public hospital in Northern Cyprus between May and November 2023. They agreed to participate in the research and were entered using the SPSS program.

### 2.8. Data Analysis

The study’s data were analyzed using SPSS (Statistical Package for the Social Sciences) for Windows 25.0 and AMOS (Analysis of Moment Structures) 23.0 programs.

“Reliability Analysis” was conducted to examine the scales’ reliability, “Explanatory Factor Analysis” was performed using the SPSS program to verify construct validity, and “Confirmatory Factor Analysis (CFA)” was performed using the AMOS program. A 27.0% sub-superordinate item analysis was applied to test the items’ discrimination.

## 3. Results

### 3.1. Content Validity

The content validity rate of the ninth item was found to be 0.875, and the value of the remaining twelve items was calculated as “1”.

### 3.2. Results of Socio-Demographic and Birth History Characteristics Analysis

53.7% of the mothers are under 28 years of age, 46.3% are aged 28 and over, 18.0% are primary school graduates, 23.9% are secondary school graduates, 37.1% are high school graduates, and 21.0% are university graduates. Most of the participants were married, and most of them were housewives. 38.5% of women have their first birth, 34.6% have their second birth, and 26.8% have their third or more births. Almost all of the mothers stated that they had no history of problematic growth, and 46.3% had a normal birth, 26.3% had emergency LSCS, and 27.3% of them gave birth by elective LSCS (Table 1).

### 3.3. Explanatory Factor Analysis Results

Before conducting exploratory factor analysis (EFA), the Kaiser-Meyer-Olkin (KMO) test was employed to assess the adequacy of the sample size for factor analysis. The analysis yielded a KMO value of 0.758, indicating that the sample was sufficiently adequate for factor analysis. KMO values between 0.50 and 1.00 are acceptable, whereas values below 0.50 suggest that the data are unsuitable for factor analysis [35]. Additionally, Bartlett’s Test of Sphericity produced a significant chi-square value (χ^2^(66) = 557.173, *p* < 0.05), further supporting the appropriateness of factor analysis for the dataset.

During the exploratory factor analysis (EFA), one item (Item 13) was excluded from the scale due to cross-loading on multiple factors (M13 = 0.494, 0.457), which hindered its clear association with a single dimension. The remaining 12 items were successfully grouped into four distinct sub-dimensions. Collectively, these factors accounted for 62.268% of the total variance. A total explained variance exceeding 50.0% in multi-factor models is generally considered acceptable and indicative of a robust factor structure [36,37].

The scale’s reliability was also evaluated using Cronbach’s alpha. The overall reliability coefficient was 0.758, indicating good internal consistency. Cronbach’s alpha values above 0.60 are typically regarded as evidence of scale reliability, suggesting that the instrument used in this study demonstrates strong internal consistency (Table 2).

### 3.4. Results of the Measurement Model of the Scale

When the correlations between variables are evaluated, it is revealed that the item factor loadings are greater than 0.40, and all correlation relationships are significant (Table 3).

### 3.5. Confirmatory Factor Analysis Results of the Scale

According to the Confirmatory Factor Analysis, the 12 items that make up the scale were related to the scale structure of the four sub-dimensions. The accepted values for fit indices in fit index calculations are shown in the table (Table 4).

### 3.6. Item Analysis Results

Table 5 presents the independent samples *t*-test results, which were conducted to evaluate each item’s discriminatory power. To assess item distinctiveness within the scale, raw scores obtained from each factor were ranked in ascending order, and the mean scores of the lower 27% and upper 27% groups were compared using an independent samples *t*-test.

The results indicate that item scores between the lower and upper groups differed significantly across all sub-dimensions (*p* < 0.05). These findings suggest that each sub-dimension of the scale demonstrates adequate discriminatory ability in measuring the intended construct (Table 5).

### 3.7. Split-Half Reliability of the Scale

The split-half reliability of the scale is 0.645.

## 4. Discussion

The primary aim of developing the Questionnaire for Assessing the Childbirth Experience (QACE) was to identify negative birth experiences among primiparous women [23]. However, each childbirth experience is unique, regardless of parity. Adverse experiences during childbirth can impact not only the mother’s physical and psychological health but also that of the newborn and the broader family unit. The literature indicates that primiparous women often experience higher levels of childbirth-related fear. In contrast, multiparous women may develop stronger fears due to previous negative birth experiences, often leading them to prefer cesarean sections [38,39,40].

After the researcher’s retrospective analysis in 2023, 871 births occurred in the clinic where data collection took place: approximately 554 were cesarean sections (LSCS), and 316 were vaginal births. The observed cesarean rate (63.6%) aligns with national trends reporting increased cesarean deliveries in Türkiye [13].

This increase may be attributed to medical indications, maternal preference, and institutional practices. Considering these trends and clinical realities, primiparous and multiparous mothers—regardless of delivery mode—were included in the study sample. This approach is consistent with similar studies conducted in culturally distinct settings such as China and France [25,27].

Demographic data revealed that 38.5% of participants were giving birth for the first time, 34.6% for the second time, and 26.8% for the third time or more. The majority of mothers reported no previous negative birth experiences. Among the participants, 46.3% had vaginal deliveries, 26.3% underwent emergency cesarean sections, and 27.3% had elective cesarean deliveries. The sampling strategy adopted in this study allows for a broader understanding of fertility behaviors and birth preferences in Turkish society. Furthermore, it facilitates exploration of how variables such as parity and delivery mode influence birth experience within a cultural context, contributing to a more comprehensive understanding of motherhood experiences.

### 4.1. Reliability Analysis

The overall Cronbach’s alpha coefficient for the scale was 0.758. Reliability coefficients range from 0 to 1, where 1 indicates no measurement error and 0 represents complete error. An alpha value of ≥0.70 is generally acceptable [41,42,43]. The alpha value obtained in this study confirms that the scale demonstrates acceptable internal consistency and reliability.

### 4.2. Exploratory Factor Analysis (EFA)

In the original QACE study, subscale reliability ranged from 0.70 to 0.84 [23], with similar ranges reported in the Iranian 0.77–0.82 [24], French 0.69–0.86 [25], and Chinese 0.611–0.844 [27] adaptations.

In this study, internal consistency coefficients for the subscales were calculated using principal component analysis and Varimax rotation: “relationship with staff” α = 0.764, “first moments with newborn” α = 0.637, “emotions during the first month postpartum” α = 0.659, and “emotional state” α = 0.571.

These findings are consistent with the reliability and validity results reported in the original and adapted versions of QACE. The highest internal consistency was found in the “relationship with staff” subscale, while the lowest was observed in “emotional state”. This may reflect the subjective and individual nature of emotional responses to childbirth. As supported by the literature, birth is a complex emotional, relational, and clinical process.

Women who experience traumatic childbirth may be more vulnerable to adverse psychological outcomes and may require specific support [44,45]. The emotional state subscale’s relatively low reliability may indicate the need for individualized support and interventions.

Before conducting EFA, the suitability of the data was assessed using the Kaiser-Meyer-Olkin (KMO) test, which yielded a value of 0.758. A KMO value between 0.50 and 1.00 indicates that the data are suitable for factor analysis [35].

One item (Item 13) was excluded during EFA due to high cross-loading across multiple factors. The remaining 12 items were grouped under four factors, which explained 62.268% of the total variance. A total explained variance exceeding 50% is considered adequate in multi-factorial scales [36,37]. The consistency of these factors was further confirmed through McDonald’s Omega coefficient, which was calculated at 0.754, indicating good internal consistency [46].

In contrast to the original QACE study, where factor congruence was weak [23], the Iranian version reported low to moderate inter-factor consistency [24]. Despite lower internal consistency in some subscales in the Turkish adaptation, the overall correlation analyses suggest that the scale’s reliability levels are adequate and acceptable.

Factor loadings in this study ranged from 0.562 (İtem 10: “The first moments with my baby were as I had imagined”) to 0.849 (İtem 11: “I am proud of myself”). Factor loadings ≥0.30 are considered significant, and those ≥0.50 are considered strong [47]. These findings indicate that the items effectively represent their respective factors. In the original QACE, loadings ranged from 0.620 (“I feel regret”) to 0.890 (“I held my baby for the first time when I wanted to”), demonstrating consistency between the Turkish version and the original structure in terms of construct validity.

This finding indicates that the minimum and maximum values obtained in the Turkish adaptation study are consistent with the scale’s original structure and support its construct validity.

In Turkish society, fertility is regarded as a unique and sacred power attributed to women, while breastfeeding is seen as an essential and sacred responsibility of motherhood. The figure of the “mother” is positioned as the source of life and birth, and has historically been revered in mythology as a sacred being who ensures the continuation of generations and symbolizes purity. Childbirth and motherhood are fundamental experiences that shape a woman’s social value and place her at the center of the Turkish family structure [47]. Although the experience of childbirth varies from woman to woman (as indicated by the low factor loading of item 10), it is perceived as a success story and a source of pride for women, regardless of whether the experience is positive or negative (as reflected in the high factor loading of item 11).

### 4.3. Confirmatory Factor Analysis (CFA)

CFA was used to evaluate the instrument’s psychometric properties and assess model fit, including construct validity, method effects, and measurement invariance. CFA values range from 0 to 1, with values closer to 1 indicating better model fit [48].

Model fit was assessed using indices such as the Root Mean Square Error of Approximation (RMSEA), Comparative Fit Index (CFI), Non-Normal Fit Index (NNFI), and Goodness-of-Fit Index (GFI). The results showed that the 12-item, four-factor structure aligns well with the hypothesized model and that all fit indices fall within acceptable thresholds (Table 4).

The results of the independent samples *t*-test to assess item discrimination are presented in Table 5. The mean scores of the lower 27% and upper 27% groups, determined based on scores from each factor, were compared. The analysis showed significant differences between the groups for all items and subscales (*p* < 0.05). This finding indicates that the subscales of the scale have sufficient power to distinguish between different levels of the measured construct.

The Spearman-Brown prophecy formula was used to assess split-half reliability. The original version of the scale included 13 items, and the calculated split-half reliability coefficient was 0.535. However, Item 13 was removed because it was highly similar to other items. After this item was excluded, the scale was reduced to 12 items, and the new analysis showed an improved split-half reliability coefficient of 0.645. This indicates that removing the item increased the internal consistency and made the scale more reliable. According to the literature, reliability coefficients of 0.60 or higher are acceptable [49]. Therefore, the coefficient of 0.645 suggests that the scale has an adequate level of internal consistency and is appropriate for use (Table 6).

## 5. Limitations of the Study

This study was conducted with Turkish mothers who gave birth in a public hospital in Northern Cyprus. Since the study was limited to a single hospital and not applied in other hospitals in Northern Cyprus or different regions of Türkiye, the generalizability of the findings is limited. This is considered a significant limitation of the research.

However, one of the study’s strengths is that the QACE scale can be applied to primiparous and multiparous mothers regardless of the mode of birth (vaginal or cesarean), as seen in the French and Chinese versions of the scale [25,27].

In addition, as also noted in the Iranian version of the scale [24], operative vaginal birth interventions (such as forceps and vacuum extraction) are now rarely performed in the clinical setting where this study was carried out (see Table 1). However, when these types of interventions are used, they may lead to more negative birth experiences for mothers [50]. In the original version of the DDDA, developed in Switzerland and France, the rate of such interventions was reported to be approximately 18.9% [23]. Therefore, the low frequency of operative interventions in the current study setting may have limited the diversity of birth experiences, another limitation of the research.

## 6. Conclusions

This study aimed to adapt the short edition of the QACE to Turkish culture and evaluate its psychometric properties. Based on the analyses and comparison results, the DDDA appears to be a culturally appropriate and promising instrument for the Turkish population, providing sufficient evidence of its strong psychometric qualities. The findings indicate that the QACE can be a reliable and valid tool for assessing mothers’ childbirth experiences. The scale should be used in future studies involving different sample designs. Furthermore, it is expected to contribute to efforts to evaluate and improve the quality of care provided during childbirth.

## Figures and Tables

**Table 1:** Socio-Demographic and Obstetric Characteristics of the Participants (*n* = 205).

Variables		*n*	%
Age	Under 28	110	53.7
($\bar{X}$ ± SS, 27.97 ± 5.63)	28 or older	95	46.3
Education	Primary School	37	18.0
	Secondary School	49	23.9
	High School	76	37.1
	University	43	21.0
Martial Status	Married	203	99.0
	Single	2	1.0
Profession	Housewife	144	70.2
	Freelance	3	1.5
	Worker	53	25.9
	Government Employee	5	2.4
Birth Count	First Birth	79	38.5
	Second	71	34.6
	Third and more	55	26.8
History of problematic birth	Stillbirth	2	1.0
	Vacuum birth	1	0.5
	Excessive bleeding	7	3.4
	No history of problematic birth	195	95.1
Mode of delivery n (%)	Spontaneous vaginal delivery	95	46.3
	Emergency LSCS (Ceserean)	54	26.3
	Elective LSCS (Ceserean)	56	27.3
Total		205	100.0

**Table 2:** Explanatory Factor Analysis Results of the Scale.

Items	Factors				Total Item Correlation
	F1: Relationship with Staff	F2: First Moments with a Newborn	F3: Feeling at 1-Month Postpartum	F4: Emotional Status	
ITEM 4	0.780	0.134	0.183	0.144	0.630
ITEM 6	0.771	0.061	−0.058	0.112	0.577
ITEM 5	0.734	0.134	0.071	0.120	0.551
ITEM 7	0.712	0.129	−0.072	0.012	0.513
ITEM 9	0.070	0.817	−0.071	0.099	0.444
ITEM 8	0.205	0.790	0.151	−0.050	0.530
ITEM 10	0.171	0.562	0.268	0.244	0.382
ITEM 11	−0.041	0.025	0.849	0.073	0.497
ITEM 12	0.066	0.190	0.787	0.123	0.497
ITEM 1	0.010	0.131	−0.100	0.798	0.349
ITEM 2	0.317	0.097	0.196	0.679	0.436
ITEM 3	0.103	−0.017	0.386	0.618	0.392
Reliability	0.764	0.637	0.659	0.571	0.758
McDonald’s Omega	0.766	0.657	0.662	0.573	0.754
Explained Variance (%)	20.347	14.390	14.035	13.496	62.268
KMO = 0.758; χ^2^(66) = 557.173; Bartlett Sphericity Test (*p*) < 0.001					

KMO: Kaiser-Meyer-Olkin.

**Table 3:** Results Regarding the Measurement Model of the Scale.

Factors	Expressions (Items)	Factor Loading	Standard Errors	t Values	*p* Values
F1: Relationship with Staff	4	0.79	-	-	-
	6	0.64	0.12	8.20	<0.05
	5	0.68	0.12	8.62	<0.05
	7	0.54	0.10	7.03	<0.05
F2: First Moments with Newborn	9	0.57	-	-	-
	8	0.70	0.24	5.57	<0.05
	10	0.56	0.20	5.31	<0.05
F3: Feelings at 1-Month Postpartum	11	0.59	-	-	-
	12	0.83	0.39	4.06	<0.05
F4: Emotional Status	1	0.42	-	-	-
	2	0.74	0.28	4.51	<0.05
	3	0.54	0.21	4.34	<0.05

**Table 4:** Goodness of Fit Values of the Structural Model of the Scale.

	Structural Model Values	Recommended Values
CMIN/DF	1.58	≤5
RMSEA	0.05	≤0.08
GFI	0.94	≥0.80
AGFI	0.90	≥0.80
CFI	0.94	≥0.80
TLI	0.92	≥0.80
IFI	0.94	≥0.80
RFI	0.81	≥0.80
NFI	0.86	≥0.80
SRMR	0.05	≤0.10

CMIN/DF: Relative Chi-Square Index. RMSEA: Root Mean Square Error of Approximation. CFI: Comparative Fit Index. AGFI: Adjusted Goodness-of-Fit Index. TLI: Tucker–Lewis Index. IFI: Incremental Fit Index. RFI: Relative Fit Index. NFI: Normed Fit Index. SRMR: Assesses the Model’s Discrepancy Based on Residuals.

**Table 5:** Results of the Factor Analysis.

Factors	Items	t (%27 Below–Above)	*p* (27% Below–Above)
F1: Relationship with Staff	Item 4	−6.83	<0.001
	Item 6	−9.38	<0.001
	Item 5	−10.53	<0.001
	Item 7	−7.08	<0.001
F2: First Moments with Newborn	Item 9	−6.01	<0.001
	Item 8	−9.52	<0.001
	Item 10	−9.80	<0.001
F3: Feeling at 1-Month Postpartum	Item 11	−4.89	<0.001
	Item 12	−3.31	<0.001
F4: Emotional Status	Item 1	−21.65	<0.001
	Item 2	−11.52	<0.001
	Item 3	−9.12	<0.001

F: Factor.

**Table 6:** The split-half reliability of the scale with 12 items.

Reliability Statistics			
Cronbach’s Alpha	Part 1	Value	0.687
		Items No.	6 *^a^*
	Part 2	Value	0.619
		Items No.	6 *^b^*
	Items		12
Correlation Between Forms			0.486
Spearman–Brown Coefficient	Equal Length		0.654
	Unequal Length		0.654
Guttman Split-Half Coefficient			0.645

(*^a^*) The items are: M1, M2, M3, M4, M5, M6; (*^b^*) The items are: M7, M8, M9, M10, M11, M12.

## Data Availability

The original contributions presented in this study are included in the article. Further inquiries can be directed to the corresponding author.
